# Differences in Radiosensitivity According to EGFR Mutation Status in Non-Small Cell Lung Cancer: A Clinical and In Vitro Study

**DOI:** 10.3390/jpm14010025

**Published:** 2023-12-25

**Authors:** Hidekazu Tanaka, Masako Karita, Kazushi Ueda, Taiki Ono, Miki Kajima, Yuki Manabe, Koya Fujimoto, Yuki Yuasa, Takehiro Shiinoki

**Affiliations:** Department of Radiation Oncology, Graduate School of Medicine, Yamaguchi University, 1-1-1 Minamikogushi, Ube 755-8505, Yamagcuhi, Japankouya@yamaguchi-u.ac.jp (K.F.); shiinoki@yamaguchi-u.ac.jp (T.S.)

**Keywords:** epidermal growth factor receptor, driver mutation, non-small cell lung cancer, radiation therapy, radiosensitivity

## Abstract

Unlike drug selection, radiation parameters (field, dose) are not based on driver gene mutations in patients with metastatic non-small cell lung cancer (NSCLC). This study aimed to compare radiosensitivity in NSCLC with and without EGFR driver gene mutations using clinical and in vitro data. The clinical study included 42 patients who underwent whole-brain radiotherapy for brain metastases from NSCLC; of these, 13 patients had EGFR mutation-positive tumors. The Kaplan–Meier method was used to calculate the cranial control rate without intracranial recurrence. In the in vitro study, colony formation and double-strand DNA breaks were examined in two EGFR mutation-negative and three EGFR mutation-positive NSCLC-derived cell lines. Colony formation was assessed 14 days after irradiation with 0 (control), 2, 4, or 8 Gy. DNA double-strand breaks were evaluated 0.5 and 24 h after irradiation. EGFR mutation-positive patients had a significantly better cranial control rates than EGFR mutation-negative patients (*p* = 0.021). EGFR mutation-positive cells formed significantly fewer colonies after irradiation with 2 or 4 Gy than EGFR mutation-negative cells (*p* = 0.002, respectively) and had significantly more DNA double-strand breaks at 24 h after irradiation (*p* < 0.001). Both clinical and in vitro data suggest that EGFR mutation-positive NSCLC is radiosensitive.

## 1. Introduction

Lung cancer is the second most common type of cancer worldwide. In 2020, the estimated number of new cases was 2,206,771 and the estimated number of deaths peaked at 1,796,144 [1]. Some lung cancer patients harbor mutations in driver genes such as EGFR, ALK, ROS1, and BRAF. EGFR driver mutations are the most common, particularly in Asians, women, and nonsmokers [2,3,4,5], and more than 40% of Japanese patients with lung adenocarcinoma are EGFR mutation-positive (EGFRm+) [6,7]. Previous reports have shown that EGFRm+ non-small cell lung cancer (NSCLC) responds well to tyrosine kinase inhibitors (TKIs) [8,9]. In NSCLC patients with distant metastasis, drug selection is based on the results of molecular testing [10,11]. However, in cases where radiotherapy is administered, molecular information is not considered when determining the irradiation field and dose.

The purpose of this study was to compare the radiosensitivity of EGFR mutation-negative (EGFRm-−) and EGFRm+ NSCLCs using clinical and in vitro data.

## 2. Materials and Methods

### 2.1. Clinical

The clinical portion of our study included patients who underwent whole-brain radiation therapy (WBRT) for brain metastases (BMs) from NSCLC at our hospital between January 2011 and March 2021. Patients with metastases from small cell lung cancer and large cell neuroendocrine carcinoma were excluded. Patient characteristics are shown in Table 1. Among the 42 enrolled patients, 28 (66.7%) were male and 14 (33.3%) were female, and the median age was 68 (33–84) years. All NSCLCs were histologically diagnosed, and 34 (81.0%) NSCLCs were adenocarcinomas. Twenty-three patients were EGFRm−, 13 were EGFRm+, and six had an unknown EGFR mutation status. The sites of specimen collection for EGFR mutation testing included the primary tumor in 20 patients, bone metastasis in five patients, lymph node metastases in three patients, brain metastases in two patients, and other sites in five patients. The reasons for the lack of EGFR testing or unknown results for the six patients were as follows. Two patients underwent surgery as the initial treatment, and pathological findings showed that there was no adenocarcinoma component. Another two patients refused drug therapy, including TKIs, but consented to receive radiation therapy. TKIs were contraindicated in one patient because of interstitial pneumonia. The remaining patient was diagnosed and received systemic therapy at another hospital and subsequently continued treatment at our hospital at his own discretion; his EGFR mutation status could not be obtained from his medical chart. The results of EGFR mutation testing were as follows: Del 19 in seven patients, L858R in five patients, and L861Q in one patient. T790M was confirmed in four cases. In addition, five other patients had been treated with TKIs prior to WBRT, and all of them became resistant to TKIs and were considered clinically resistant. Cases in which EGFR mutation status was not investigated were treated as EGFRm−. To address concerns regarding the classification of patients with unknown EGFR mutations as EGFRm−, an additional analysis was conducted for 36 patients, excluding the six patients with unknown EGFR mutations. Most patients received a radiation dose of 30 Gy in 10 fractions. BMs were detected in 14 (33.3%) patients at the time of diagnosis of NSCLC, and these patients received WBRT. Six (14.3%) patients had recurrence of BMs after the initial definitive therapy, and they received WBRT. BMs were detected or worsened in 22 (52.4%) patients during the course of treatment of stage IV NSCLC, and these patients received WBRT. Almost all patients (90.5%) had three or more brain metastases; the maximum size of the brain metastases was 15 (4–55) mm. Almost all patients (92.9%) had distant metastases in addition to brain metastases. Nine patients (21.4%) received one or more TKIs after WBRT; the TKIs administered were erlotinib (six cases), osimertinib (four cases), and gefitinib (one case). Nineteen of 42 patients (45.2%) received some form of chemotherapy after whole brain irradiation. Chemotherapy regimens, including duplicates, were as follows: docetaxel for six patients, carboplatin + pemetrexed for five patients, S-1 for three patients, vinorelbine for three patients, and other regimens for nine patients.

Intracranial recurrence was defined as the growth of at least one brain metastasis or the appearance of a new brain metastasis after WBRT. The Kaplan–Meier method was used to calculate the cranial control (CC) rate without intracranial recurrence. Comparisons between two groups were performed using the log-rank test. A *p*-value < 0.05 was considered to indicate a statistically significant difference.

### 2.2. In Vitro

In the in vitro study, five NSCLC-derived cell lines were used: two were EGFRm− (A549 and VMRC-LCD) and three were EGFRm+ (HCC4006, PC-9, and NCI-H1975). HCC4006 and PC-9 cells have EGFR mutations in exon 19 (mainly deletions), and NCI-H1975 cells have EGFR mutations in exon 21 (mainly L858R point mutations) [12,13]. For colony formation assays, cells seeded at approximately 50% confluence were irradiated with 2, 4, or 8 Gy or left unirradiated (control). Irradiation was performed using a TrueBeam STx radiotherapy system (Varian Medical Systems, Palo Alto, CA, USA), with 4 MV X-rays at 250 MU/min. A 10 mm bolus was placed on each culture dish before irradiation. After irradiation, 1000 cells in each dish were reseeded and cultured for 14 days. Colonies were then stained with crystal violet and counted. The ratio of the number of colonies in each dose group to the number of colonies in the control group was compared between EGFRm− and EGFRm+ cells. Comparisons were made using the Mann–Whitney U test.

In addition, cells were seeded in six-well plates at approximately 70% confluence and irradiated with 4 Gy. DNA double-strand breaks (DSBs) were evaluated using γH2AX as a surrogate marker at 0.5 and 24 h after irradiation. Fluorescent immunostaining was performed using anti-gamma H2AX (phospho S139) antibody (ab11174) (abcam, Cambridge, UK) as the primary antibody, goat anti-rabbit IgG H&L (Alexa Fluor 488) (ab150077) (abcam, Cambridge, UK) as the secondary antibody, and DAPi (PureBlu DAPI Nuclear Staining Dye #1351303) (BIO-RAD, Hercules, CA, USA) for nuclear staining. The number of γH2AX foci at each time point was compared between the EGFRm− and EGFRm+ cell lines, and differences were assessed using the *t*-test. A *p*-value < 0.05 was considered to indicate a statistically significant difference.

## 3. Results

### 3.1. Clinical

The median follow-up period was 4 (range, 1–35) months. Intracranial recurrence was observed in 14 (33.3%) patients during the follow-up period. Thirty-nine (92.9%) patients died during the follow-up period.

**Figure 1:**
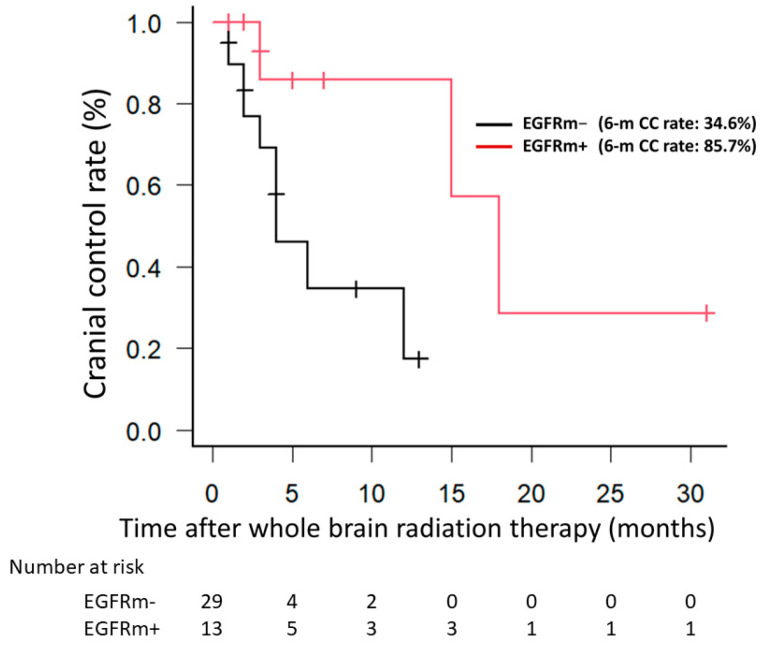
The Kaplan–Meier method was used to calculate the cranial control rate without intracranial recurrence. EGFRm+ patients had a significantly better cranial control rate than EGFRm− patients. EGFRm+, EGFR mutation-positive; EGFRm−, EGFR mutation-negative.

EGFRm+ patients had a significantly better CC rate than did EGFRm− patients (*p* = 0.021) (Figure 1). The same analysis was performed after excluding the six patients with an unknown EGFR status, and the results revealed a significantly better CC rate for EGFRm+ patients than for EGFRm− patients (*p* = 0.014).

**Figure 2:**
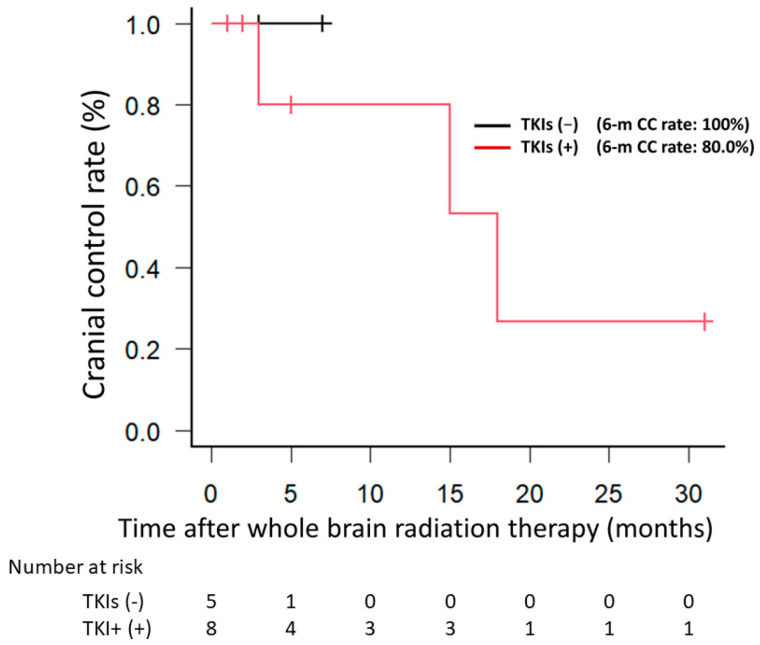
When only EGFRm+ patients were examined, cranial control rates did not differ significantly between EGFRm+ patients treated with and without TKIs. EGFRm+, EGFR mutation-positive; TKIs, tyrosine kinase inhibitors.

**Figure 3:**
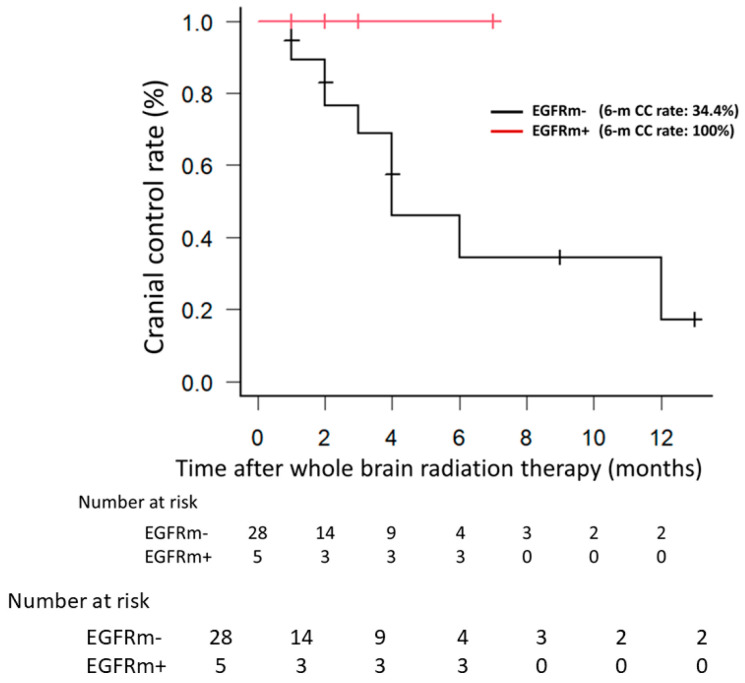
When only patients who did not receive TKIs were examined, EGFRm+ patients showed a trend toward better CC rates compared to EGFRm− patients. No intracranial recurrence was observed in EGFRm+ cases. EGFRm+, EGFR mutation-positive; EGFRm−, EGFR mutation-negative; TKIs, tyrosine kinase inhibitors.

The CC rate did not differ significantly between EGFRm+ patients who received TKIs and those did not receive TKIs (*p* = 0.527) (Figure 2). When only patients who did not receive TKIs were examined, the EGFRm+ group showed a trend toward a better CC rate compared with the EGFRm− group (*p* = 0.168) (Figure 3).

### 3.2. In Vitro

**Figure 4:**
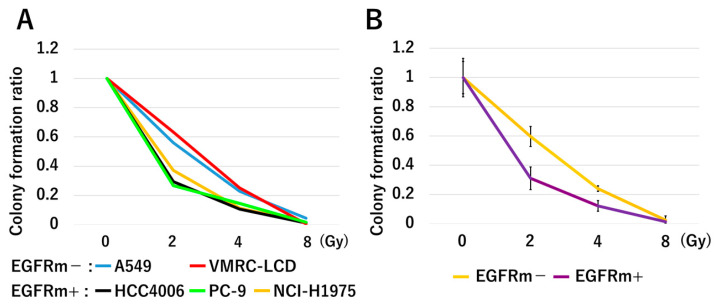
(**A**) Colony formation ratio (number of colonies in each dose group/number of colonies in the unirradiated control) for each cell line. (**B**) Colony formation ratio for the EGFRm− and EGFRm+ cell lines. EGFRm+, EGFR mutation-positive; EGFRm−, EGFR mutation-negative.

The number of colonies formed after irradiation with 2 or 4 Gy was significantly lower in EGFRm+ cells than in EGFRm− cells (*p* = 0.002, respectively) (Figure 4). The number of colonies formed after irradiation with 8 Gy was unaffected by EGFR mutation status (*p* = 0.767).

**Figure 5:**
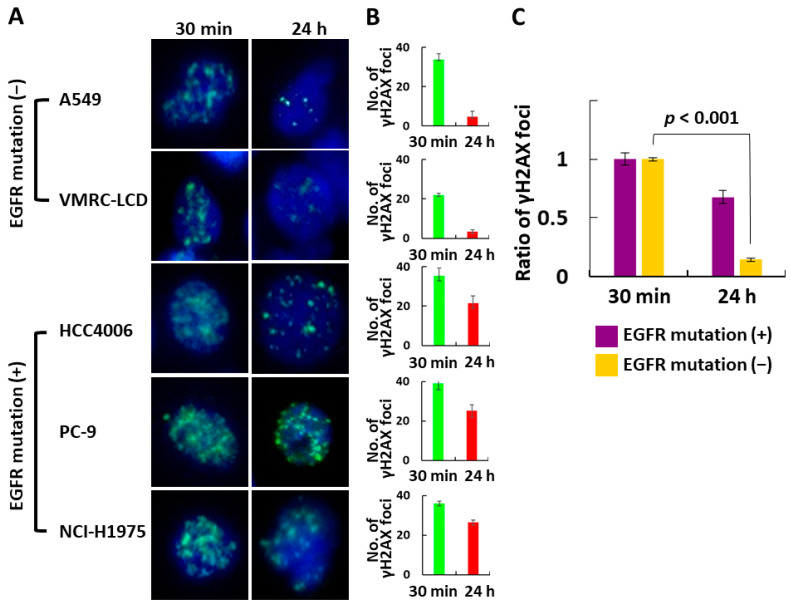
(**A**) Fluorescence immunostaining of γH2AX (green), a surrogate marker of DNA double-strand breaks, in each cell line. (**B**) Number of γH2AX foci in each cell line. (**C**) Number of γH2AX foci in the EGFRm− and EGFRm+ cell lines. EGFRm+, EGFR mutation-positive; EGFRm−, EGFR mutation-negative.

EGFRm+ cells had significantly more DNA DSBs at 24 h after irradiation than EGFRm− cells did (*p* < 0.001) (Figure 5).

## 4. Discussion

Clinical studies have shown that the local control rate is significantly higher in EGFRm+ patients than in EGFRm− patients after definitive chemoradiotherapy for locally advanced NSCLC [14,15,16]. Moreover, several reports suggest that brain metastases from EGFRm+ NSCLCs are more radiosensitive than are those from EGFRm− NSCLCs [17,18,19]. Similarly, in the present study, the six-month CC rate after WBRT was significantly higher in EGFRm+ patients than in EGFRm− group patients (85.7% vs. 34.6%). However, EGFRm+ patients are known to respond well to TKIs, and nine (21.4%) patients in our study had received TKIs. Although there was concern that the TKI usage might have influenced our results, two additional comparisons indicated that this was not the case. First, when only EGFRm+ patients were examined, the CC rate did not differ significantly between those who did vs. did not receive TKIs. Second, when only patients who did not receive TKIs were examined, although the CC rate of EGFRm+ patients tended to be better than that of EGFRm− patients, this difference was not statistically significant. Nevertheless, no intracranial recurrence was observed in EGFRm+ cases, which we consider a meaningful result. To eliminate the possibility that the inclusion of patients with unknown EGFR mutations may have affected the results, the same analysis was performed after excluding the six patients with an unknown EGFR status, and the results revealed a significantly better CC rate for EGFRm+ patients than for EGFRm− patients (*p* = 0.014).

Autophosphorylation of tyrosines in the intracellular domain of the epidermal growth factor receptor (EGFR) activates downstream tyrosine kinases and signaling pathways involved in tumor growth, survival, angiogenesis, invasion, and metastasis [20,21,22]. EGFR overexpression has been shown to increase the radiosensitivity of various cancer types [23,24,25,26,27], and EGFRm+ NSCLC cell lines are highly radiosensitive in vitro [28,29]. In agreement, we found that EGFRm+ cells formed significantly fewer colonies after irradiation with 2 or 4 Gy than did EGFRm− cells. However, there was no significant difference after irradiation with 8 Gy, presumably because of the very small number of colonies formed regardless of the EGFR mutation status. EGFRm+ cells had significantly more DNA DSBs 24 h after irradiation than EGFRm− cells did. This suggests that most DSBs were repaired within 24 h after irradiation in the absence vs. the presence of EGFR mutations.

Immunotherapy is now a new treatment option. Radiation therapy is compatible with immunotherapy. There have been reports of cases in which an abscopal effect occurred when radiotherapy and immunotherapy were used together [30]. It has also been reported that a combination of radiotherapy and immunotherapy improves the treatment efficacy [31,32,33]. It is believed that radiotherapy increases antigen presentation, which enhances the effectiveness of subsequent immunotherapy [34,35,36,37,38]. Thus, radiotherapy followed by immune checkpoint inhibitor treatment is a good option for EGFRm− cases. On the other hand, for EGFRm+ cases, TKI is obviously the first choice; however, when radiation therapy is performed, especially WBRT, it might be possible to reduce the dose by considering the radiosensitivity. Cognitive decline due to WBRT is an important and concerning adverse event [39]; however, it can be prevented if the dose can be reduced for EGFRm+ patients. In recent years, hippocampal-avoidance WBRT to prevent cognitive de-cline has been a hot topic [40,41], although no evidence-based conclusion regarding the feasibility of reducing the dose around the hippocampus has been reached. The results of our study suggest that EGFRm+ patients might be a select group of patients for whom hippocampal-avoidance WBRT is acceptable. Stereotactic radiation therapy is also an important option for BMs [42,43], although it involves the risk of radionecrosis because high doses are administered [44,45]. If the dose required for tumor control in EGFRm+ patients is found to be lower than the conventionally used dose, the risk of radionecrosis may be reduced. Furthermore, stereotactic body radiation therapy for primary lung tumors has the potential for dose reduction in terms of radiation therapy alone. This may be the first step toward personalized radiation therapy in which the radiation dose is adjusted according to the presence or absence of driver mutations. It may be possible to adjust the dose by reducing the dose for EGFRm+ patients or increasing the dose for EGFRm− patients. This has also been discussed in the revised manuscript.

## 5. Conclusions

In conclusion, both in vitro and clinical data suggest that EGFRm+ NSCLC cells are radiosensitive. In the future, precision radiotherapy, such as dose prescription or field management, may be realized based on the status of EGFR driver mutations in patients with metastatic NSCLC.

## Figures and Tables

**Table 1:** Characteristics of the patients (N = 42).

Characteristics	Description	Results
Sex	Male/female	28 (66.7)/14 (33.3)
Age (years)		68 (33–84)
Pathology	Adenocarcinoma	34 (81.0)
	Squamous cell carcinoma	4 (9.5)
	Pleomorphic canrcinoma	1 (2.4)
	Large cell carcinoma	1 (2.4)
	NSCLC, NOS	2 (4.8)
EGFR mutation	Positive/negative	13 (31.0)/29 (69.0)
Performance status	0	5 (11.9)
	1	20 (47.6)
	2	9 (21.4)
	3	6 (14.3)
	4	2 (4.8)
Irradiated dose	30 Gy in 10 fractions	36 (85.7)
	40 Gy in 20 fractions	4 (9.5)
	37.5 Gy in 15 fractions	2 (4.8)
Primary site	Controlled/uncontrolled	16 (38.1)/26 (61.9)
Extracranial disease	Presence/absence	39 (92.9)/3 (7.1)
Number of BMs	1	2 (4.8)
	2	2 (4.8)
	≥3	38 (90.5)
Maximum size of BM (mm)		15 (4–55)
Use of TKIs after WBRT	Yes/no	9 (21.4)/33 (78.6)
STI	Before/after WBRT	9 (21.4)/2 (4.8)
Surgery	Before/after WBRT	2 (4.8)/0 (0)

All results are presented as N (%), excepting age and maximum size of BM, which are presented as median (range). NSCLC, non-small cell lung cancer; NOS, not otherwise specified; BM, brain metastasis; WBRT, whole brain radiation therapy; TKIs, tyrosine kinase inhibitors; STI, stereotactic irradiation.

## Data Availability

The datasets used and/or analyzed during the current study are available from the corresponding author on reasonable request.
